# Burden of carpal tunnel syndrome and its associated factors among construction industry workers in Gondar town, Ethiopia

**DOI:** 10.3389/fpubh.2024.1365124

**Published:** 2024-06-12

**Authors:** Nigus Bicha, Moges Gashaw, Samuel Teferi Chanie, Melisew Mekie, Ermias Solomon Yalew

**Affiliations:** ^1^Physiotherapy Unit, Boru Meda Hospital, Amhara Health Bureau, Dessie, Ethiopia; ^2^Department of Physiotherapy, College of Medicine and Health Sciences, University of Gondar, Gondar, Ethiopia; ^3^Discipline of Physiotherapy, Graduate School of Health, Faculty of Health, University of Technology Sydney, Sydney, NSW, Australia

**Keywords:** carpal tunnel syndrome, construction workers, work-related musculoskeletal disorder, Ethiopia, industry workers

## Abstract

**Background:**

Carpal tunnel syndrome is characterized by symptoms such as pain, numbness, or tingling on the anterior surface of the index, middle, or radial half of the ring finger, which is frequently associated with weakness of hand grip, and nocturnal pain and/or numbness resulting from compression of the median nerve at the carpal tunnel between the carpal bones and the transverse ligament. The construction industry involves many activities aside from the building process, such as landscaping, painting, electrical supply, plastering, paving, and telecommunications. Performing such tasks involves repetition of wrist flexion and extension, forceful grip with the hand, and/or vibrations of the hand and arm. This study aimed to assess the prevalence of carpal tunnel syndrome and its associated risk factors among construction workers.

**Method:**

An institutional-based cross-sectional study design was conducted among six construction sectors in Gondar from April to July 2021. An interviewer-administered questionnaire was prepared from the literature with a Katz hand diagram, and a physical examination and a special test (carpal compression test, Phalen’s, and Tinel’s test) were carried out for those participants who reported pain during the interview. Binary logistic regression was conducted with SPSS 25 to identify the associated risk factors for carpal tunnel syndrome. The strength of the association was detected by the adjusted odds ratio.

**Result:**

A total of 333 study participants aged 18–70 years were included in this study. Among the participants, 11.7% (AOR: 95%CI: 8.1–15.3) had carpal tunnel syndrome. Age, cigarette smoking, work experience, and working with finger-pressing tools were risk factors significantly associated with carpal tunnel syndrome among construction workers with a *p*-value of <0.05.

**Conclusion:**

The magnitude of carpal tunnel syndrome was 11.7% among construction workers. Being of older age, having more experience, cigarette smoking, and working with finger-pressing tools were risk factors significantly associated with carpal tunnel syndrome among construction workers. Employers should implement work safety education programs that raise awareness about the risks of cigarette smoking and encourage employers and supervisors to seek early medical intervention and treatment for carpal tunnel syndrome before it becomes a chronic problem.

## Background

Work-related musculoskeletal disorders are a main cause of productivity loss at work, functional impairments, and permanent disability (1). Chronic musculoskeletal stresses resulting from strained postures or repetitive, forceful movements can cause chronic musculoskeletal conditions such as tendinitis, epicondylitis, carpal tunnel syndrome, and low back pain (2).

Carpal tunnel syndrome (CTS) is characterized by symptoms such as pain, numbness, or tingling on the anterior surface of the index, middle, or radial half of the ring finger, which is frequently associated with weakness of hand grip, and nocturnal pain and/or numbness resulting from compression of the median nerve at the carpal tunnel formed by the carpal bones and the transverse ligament (3, 4). The boundaries of the carpal tunnel include the carpal bones (posteriorly, medially, and laterally) and the transverse carpal ligament, i.e., flexorum retinaculum, anteriorly (5).

The construction industry involves many activities aside from the building process, such as landscaping, painting, electrical supply, plastering, paving, and telecommunications (6). Performing such tasks involving repetition of wrist flexion and extension, forceful grip with the hand, and/or vibrations of the hand and arm induced by hand-held vibratory tools can damage the median nerve and cause CTS (7). In addition, construction workers work in the same position for long periods, suffer overexertion due to carrying and lifting heavy objects, and engage in repetitive tasks. The frequency of the task and the percentage of the time spent on the repetitive tasks are important. Awkward body postures and whole-body vibrations in the construction industry are high-risk factors for CTS and other upper extremity musculoskeletal disorders (8).

Carpal tunnel syndrome is a common clinical concern, with annual incidence rates of 0.5–5.1 per 1,000 in the general population and 6–15 in industry workers (9). An 11-year follow-up research showed that the prevalence of CTS among industrial workers was approximately 13% (10). In a study performed in the United States, the prevalence of CTS among construction apprentice workers was 8.2% (11). According to another study performed in the United States, up to 10% of construction apprentice workers suffer from CTS (12). A cross-sectional study performed in Italy reports that the prevalence of CTS is 14.1% (3). The prevalence of CTS is 3.8% in the general Finnish population and is 2.5 times more common in women than in men (13). A cross-sectional study conducted in Iran found that the prevalence of CTS was 11.9%. In Africa, according to the findings of a study conducted in Egypt, 27.6% of construction workers suffered from CTS with the frequency of symptoms in the following order: hand/wrist pain (93.8%), numbness (65.6%), and nocturnal exacerbations (56.3%) (14). A cross-sectional study performed in Ethiopia among bankers revealed that 11.7% of bankers suffered from CTS (15).

Previous epidemiological studies revealed that sex, older age, having higher BMI, smoking cigarette, dominant handedness, type of work, speed, movement repetition, bending and twisted, and work experience are related to CTS (12, 14, 16). There is a lack of data about the prevalence and associated factors of CTS among construction workers in Ethiopia. Therefore, the present study aims to contribute to this area of research by determining the magnitude and factors related to CTS among building construction workers in Gondar, Ethiopia.

## Methods and materials

### Study design and setting

An institutional-based cross-sectional study was conducted among six construction sectors in Gondar town from April to July 2021.

The town is located 738 km northwest of Addis Ababa, the capital of Ethiopia. It has a population of 206,987 (17) and has six construction sites, namely, Unity, Rama, Zamra, Siyum, Yirgalem, and Alinando. All construction companies had 510 workers engaged in different working units in all sites.

### Source population, study population, and inclusion and exclusion criteria

Adult construction workers who were actively working at construction sites were enrolled in the study. This study included six selected adult construction workers aged at least 18 years who were actively involved in construction and provided informed consent. However, construction workers who had wrist trauma, who had diabetes mellitus, who had known pregnancy, were staff office workers, and who had been working for >1 year were excluded from the study.

### Sample size and sampling procedure

The sample size was based on single population proportion formula a sample size was determined; 95% level of significance the proportion of assumptions or expected frequency 27% prevalence of carpal tunnel syndrome done in Africa Egypt marginal error of 5%. Based on the job description, the study populations were stratified into six different strata. The number of samples from each stratum was determined using the proportional allocation formula. Finally, a simple random sampling technique was employed to select 348 samples from the strata.

$n=(z\;\left(/2\right)2\;\left(p\right)\;\left(1-p\right)\;d2$

$n=\left(1.96\right)2\;\left(0.27)\;(1-0.27\right)=302.87$

(0.05)2 *n* = 303

*n* = 302.87 ≈ 303,

Taking into consideration a 15% non-response rate from *n* = 303×15/100 = 45, the final *n* = 348.

A total of 137, 110, 21, 35, 18, and 27 participants from Unity, Rama, Zamra, Siyum, Yirgalem, and Alinando, respectively, were proportionally allocated for each construction site. Then, a simple random sampling technique was used to recruit the actual number of study participants.

### Data collection instrument

An interviewer-administered questioner was prepared from the literature, and physical examination was performed to gather information from the participation. Carpal tunnel syndrome was assessed through symptoms reported on the Katz hand diagram and physical examination. Durkan’s compression test, the flexion and compression test, Phalen’s test, and Tinel’s test were performed for all participants.

Construction workers are defined as workers involved in many types of activities aside from the building process, such as landscaping, painting, electrical supply, plastering, paving, and telecommunication.

Durkan’s or carpal compression test consists of applying direct pressure on the carpal tunnel and the underlying median nerve. This task is performed by an examiner who exerts even pressure, with both thumbs, to the median nerve in the carpal tunnel. The examiner presses the thumbs over the carpal tunnel and holds the pressure for 30 s. An onset of pain or paresthesia in the median nerve distribution within 30 s is a positive result of the test (18).

The flexion and compression test was performed with the elbow extended, the forearm in supination, and the wrist flexed to 60°. Even and constant digital pressure was then applied with one thumb over the median nerve at the carpal tunnel. The time before the development of paresthesia or numbness in the distribution of the median nerve was recorded, and the test was considered positive if symptoms occurred within 30 s (19).

The wrist flexion test (Phalen’s test) is positive when, after full flexion of the wrist for up to 60 s, the patient experiences symptoms of paresthesia or numbness within the distal territory of the median nerve, similar to those of which he complains during the night or aggravation of the existing paresthesia if they are present permanently (20, 21).

Tinel’s test involves a tap over the median nerve as it passes through the carpal tunnel in the wrist A positive response is defined as a sensation of tingling in the distribution of the median nerve over the hand (21).

The Katz Hand diagram is a self-administered hand diagram for the diagnosis of carpal tunnel syndrome, including symptoms such as tingling, numbness, or decreased sensation with or without pain in at least two of digits 1, 2, or 3, as well as wrist pain or radiation proximal to the wrist, but excluding symptoms in the palm and dorsum of the hand (22).

Independent variables such as sociodemographic variables and repetitive wrist movement, especially when the self-reported time spent in activities with the wrist flexed or extended was 20 h/week (23). Working with hands above the shoulder level is defined as working with hands above the shoulder level for at least 1 h (24).

Smoker: Individuals who reported smoking at least one cigarette daily will be considered smokers.

Working with vibratory tools is defined as working with a hand-vibratory tool for at least 2 h (24).

Body mass index can be measured as weight in kilogram divided by square of height in meters (kg/m2). Based on the BMI, the workers were categorized as under-weight (<18.5 kg/m^2^), normal (18.5–24.99 kg/m^2^), overweight (>25–29.9 kg/m^2^), and obesity (>30 kg/m^2^) (25).

Awkward posture is defined as using bending or twisted posture during work (26).

Alcohol Drinking is defined as the consumption of any kind of alcohol at least for two times per week for different purposes.

### Data quality assurance

To ensure the quality of the data, the data collectors and supervisors were trained for 1 day before the actual data collection process on how to approach the participants, the objective of the study, and ethical issue. The data collection tool was pretested before the actual data collection process to check for the accuracy of responses, language clarity, and appropriateness of the tools. The questionnaire was pretested by taking 5% (17) of the sample population, and we found that the interclass correlation coefficient (ICC) value of the tool was 0.86. The supervisor checked for the completeness and consistency of the data.

### Data processing and analysis

The collected data were checked for completeness and clarity, coded, and then entered in EpI data version 4.6.0.4 and analyzed by using SPSS version 25. Descriptive, bivariate, and multivariate analyses had been used with a *p*-value <0.2 in the bivariate model further analyzed using the multivariate model. A logistic regression analysis was used to control possible confounders and to examine the association between different independent variables. The strength of the associations was determined using an adjusted odds ratio (AOR) with a 95% confidence interval (CI) with a *p*-value of less than 0.05. The model fitness was checked using the Hosmer and Lemeshow test (0.865).

## Results

### Sociodemographic characteristics of the participant

A total of 333 study participants were included in this study with a response rate of 95.7%; the reasons for non-responses were lack of time and lack of interest. The age range of the study participants was between 18 and 70 years; a majority of the study participants (217) (65.2%) were found in the age range of <30 years old, and the mean age of the participants was 30.83 years with a standard deviation of 9.183. Men included the majority of the participants (219) (65.8%), and women composed 114 of the participants (34.2%). More than half of the participants (66.7%) were urban dwellers, and 183 (55%) participants were married. The education level of most participants (125) (37.6%) were complete primary school, and 319 (95.8) are right-handed. In addition, 269 (80.8%) participants had normal body mass index (18.5–24.99), out of which 53 (15.9%) were underweight (Table 1).

**Table 1:** Sociodemographic characteristics of carpal tunnel syndrome in construction workers in Gondar, Ethiopia, June 2021 (*n* = 333).

Variables	Frequency (*n*)	Percentage (%)
Sex		
Male	219	65.8
Female	114	34.2
Age in years		
<30	217	65.2
30–45	89	26.7
≥46	27	8.1
BMI		
Underweight (<18.5)	53	15.9
Normal (18.5–24.99)	269	80.8
Overweight (>25–29.9)	11	3.3
Marital status		
Married	183	55
Single	150	45
Level of education		
No formal education	83	24.9
Complete primary school	125	37.5
Complete secondary school	93	27.9
Diploma/degree	32	9.6
Residence		
Rural	111	33.3
Urban	222	66.7
Handedness		
Right	319	95.8
Left	14	4.2

### Behavior characteristics of carpal tunnel syndrome in construction workers in Gondar Ethiopia, June 2021, (*n* = 333)

A total of 92 (27.6%) study participants consumed alcohol, and most of them, i.e., 296 (88.9%) of them, were non-smokers (Table 2).

**Table 2:** Behavior characteristics of carpal tunnel syndrome in construction workers.

Variables	Frequency (*n*)	Percentage (%)
Alcohol consumption		
Yes	92	27.6
No	241	72.4
Cigarette smoking		
Yes	37	11.1
No	296	88.9
Regular exercise		
Yes	26	7.8
No	307	92.2

### Work-related characteristics of carpal tunnel syndrome in construction workers in Gondar Ethiopia, June 2021, (*n* = 333)

Most participants had a duration of employment of less than 5 years (2,430) (73%), and the highest percentage of participants include daily wage laborers (118) (35.4%), carpenters (76) (22.8%), and only 10 (3%) were painters. In addition, 236 (70.9%) participants did not work with vibratory tools, and most of them had experienced bending and twisting of the hand (322) (96.7). Most of the participants (289) (86.8%) used their wrist repetitively, and 289 (86.8%) workers had used their hand to perform activities requiring involvement above shoulder level, and 278 (83.5) performed load carrying manually (Table 3).

**Table 3:** Work-related characteristics of carpal tunnel syndrome in construction workers.

Variables	Frequency (*n*)	Percentage (%)
Work experience		
<5 years	243	73
>5 years	90	27
Occupational history		
Carpenter	76	22.8
Painter	10	3
Daily labor worker	118	35.4
Masonry	39	11.7
Electrical	12	3.6
Plasterer	42	12.6
Other	36	10.8
Working with vibratory tools		
Yes	97	29.1
No	236	70.9
Bend and Twist		
Yes	322	96.7
No	11	3.3
Hands above shoulder		
Yes	289	86.8
No	44	13.2
Carrying load		
Yes	278	83.5
No	55	16.5
Repetitive moments on the hand		
Yes	230	69.1
No	103	31.9
Working with finger pinching tools		
Yes	29	8.7
No	304	91.3
Working with finger pressing tools		
Yes	40	12.0
No	293	88.0

### Prevalence of carpal tunnel syndrome among construction workers

**Figure 1:**
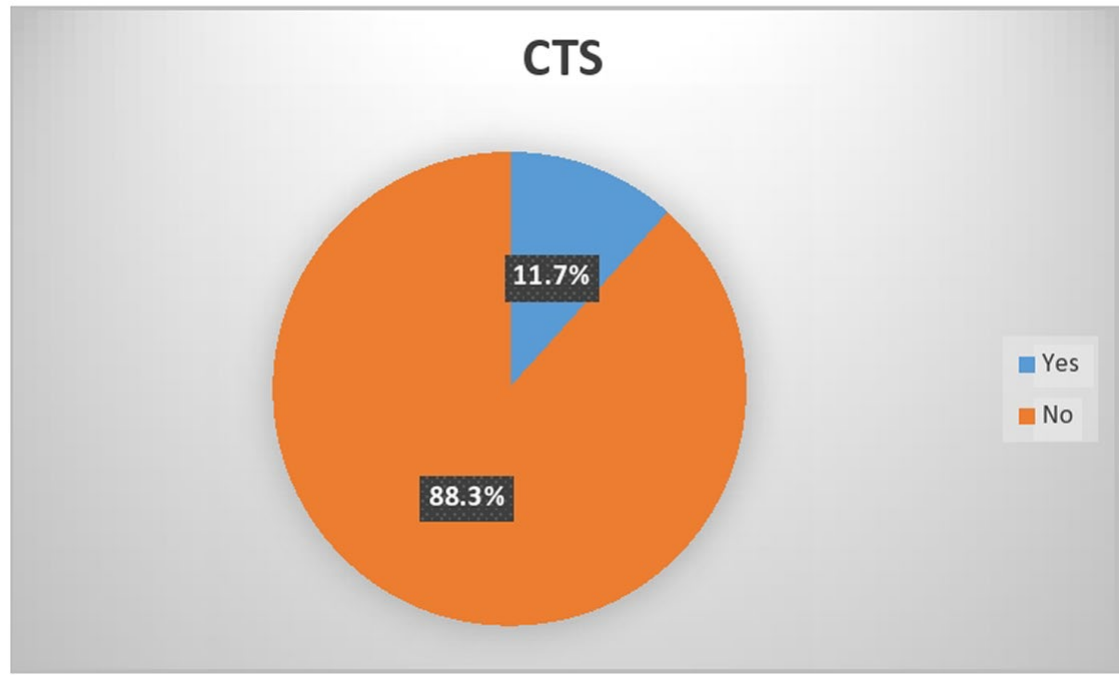
Prevalence of carpal tunnel syndrome among construction workers.

Among all 333 study participants, the annual prevalence of carpal tunnel syndrome was 11.7% with 95% CI, as shown in Figure 1. The prevalence of carpal tunnel syndrome was significantly higher (*n* = 30; 13.7%) among men and those who had a normal body mass index (*n* = 29; 10.8%), and in those with no formal education (*n* = 18; 21.7%). In terms of residence, among 222 urban residents, 26 (11.7%) had carpal tunnel syndrome. According to hand dominance, 39 (12.9%) were right-handed positive, 280 (87.8%) were right-handed negative, 14 were left-handed positive, and 100% were left-handed negative; in terms of those who consumed alcohol, 17 (18.5%) had CTS; and in terms of those who were cigarette smokers, 13 (35.1%) had CTS. Regarding the part of the hand affected, the right hand was affected in 20 (51.6%), the left hand in 7 (17.9%), and both hands in 12 (30.8%).

### Factors associated with carpal tunnel syndrome among construction workers

A binary logistic regression analysis was executed on each of the factors that were found to be significantly associated with the prevalence of carpal tunnel syndrome among construction workers. Age, sex, marital status, alcohol consumption, cigarette smoking, work experience, working with vibratory tools, having repetitive movements on the hand, working with finger-pressing tools, and working with finger-pinching tools were significantly associated with the prevalence of carpal tunnel syndrome among construction workers at a *p*-value of <0.2 in the bivariable logistic regression. Those variables were included in the multivariable logistic regression model and further analyzed to adjust for potential confounders and identify predictors of the prevalence of carpal tunnel syndrome among construction workers. In a multivariable logistic regression analysis, age, cigarette smoking, work experience, and working with finger-pressing tools (tools) were variables significantly associated with the prevalence of carpal tunnel syndrome among construction workers.

A construction worker over the age of 45 years are at 3.723 times odds of developing carpal tunnel syndrome compared with construction workers under the age of 30 years [AOR: 3.723, CI 95% (1.085–12.776)]. Individuals who smoke cigarettes had a 3.19-fold increased risk of developing carpal tunnel syndrome compared to construction workers who had no such history of cigarette smoking [AOR: 3.197; 95% CI: (1.153–8.866)]. Those workers who had a work experience greater than or equal to 5 years are 6.5 times more likely to develop carpal tunnel syndrome compared to those with less than 5 years of work experience [AOR: CI, 95% 6.532 (2.488–17.144)]. Individuals working with finger-pressing materials or tools are at 4.575 times odds of having carpal tunnel syndrome compared with construction workers not working with finger-pressing materials or tools [AOR: CI, 95% 4.574 (1.230–17.010)] (Table 4).

**Table 4:** Factors associated with carpal tunnel syndrome among construction workers.

Variables	Carpal tunnel syndrome		Univariate COR (95%CI)	Multivariate AOR (95%CI)	*p*-value
	No	Yes			
Age in years					
<30	203	14	1	_	
31–45	76	13	2.480 (1.115–5.518)	1.454 (0.539–3.921)	0.460
>45	15	12	11.600 (4.565–29.474)	3.723 (1.085–12.776.)	0.037^*^
Sex					
Male	189	30	1.852 (0.847–4.049)	0.454 (0.163–1.269)	0.132
Female	105	9	1	_	
Marital status					
Married	156	27	1.990 (0.971–4.079)	0.735 (0.288–1.875)	0.519
single	138	12	1	_	
Alcohol consumption					
Yes	75	17	2.256 (1.377–4.476)	1.554 (0.640–3.774)	0.330
No	219	22	1	_	
Cigarette smoking					
Yes	24	13	5.628 (2.564–12.343)	3.197 (1.153–8.866)	0.026^*^
No	270	26	1	_	
Work experiences in years					
>5	232	11	1		
> = 5	62	28	9.525 (4.492–20.197)	6.532 (2.488–17.144)	0.000^***^
Working with vibratory tools					
Yes	80	17	2.067 (1.044–4.93)	1.222 (0.508–2.940)	0.654
No	214	22	1	_	
Repetitive moments on the hand					
Yes	196	34	3.400 (1.289–8.965)	3.125 (0.975–10.015)	0.055
No	98	5	1	_	
Working with finger pressing tool					
Yes	29	11	3.590 (1.620–7.956)	4.574 (1.230–17.010)	0.023^*^
No	265	28	1	_	
Working with finger pinching tool					
Yes	21	8	3.355 (1.371–8.211)	0.986 (0.225–4.312)	0.985
No	273	31	1	_	

1 = reference category; AOR, adjusted odds ratio; CI, confidence interval; COR, crude odds ratio; * = statistically significant at a *p*-value of < 0.05.

## Discussion

The present study aimed to contribute to the research in determining the magnitude and factors related to CTS among 333 building construction workers in Gondar, Ethiopia. Our study results revealed that, out of the total participants, 39 (11.7%) experienced CTS. The prevalence of carpal tunnel syndrome was significantly higher (13.7% *n* = 30) among men. According to handedness and the prevalence and distribution of CTS among the 39 carpal tunnel syndrome construction workers, 71.8% of workers diagnosed with CTS had pain, 59% had tingling, and 71.8% of them experienced numbness and 25.6% experienced decreased sensation. In addition, the highest prevalence of CTS was among electricians (33.3%), followed by painters (30%). The lowest was among daily labor workers (5.1%). In terms of the affected hand, the right hand was affected in 20 (51.6), the left hand in 7 (17.9%), and both hands in 12 (30.8%) participants.

Our study’s prevalence rate (11.7%) is similar to the prevalence rate found in a cross-sectional study conducted in Iran, with a prevalence rate of 11.9% (27). In the US, carpal tunnel syndrome (CTS) affects up to 10% of construction apprentices (12), and in a cross-sectional study conducted in Germany, the prevalence of CTS in working populations was 10.9%, and a cross-sectional study conducted in Italy reported that the prevalence of CTS was 14.1% (3). The reason for the abovementioned prevalence rates could be the that most of the industrial workers are prone to different activities such as carrying load, having repetitive movements on the hand, and working with vibratory tools. Repetition of wrist flexion and extension forceful grip with the hand and/or vibrations of the hand and arm induced by hand-held vibratory tools are the greatest and the most accepted occupational risk factors that can damage the median nerve and cause CTS (16).

In contrast, the prevalence of CTS among construction workers in our study was higher than the prevalence of CTS among construction workers based on national surveys. The prevalence of CTS among construction workers determined in this study is much higher than that in the 1988 National Health Interview Survey (Occupational Health Supplement) involving 30,090 “recent workers,” which revealed a 1-year period prevalence of 2.12% for self-reported CTS in the construction industry (16).

In addition, the estimated prevalence rates for CTS have been 1–5% in the general population owing to the fact that the general population may be less experienced with the work load that is common to the construction industry. Since most of the workers in the construction industry are prone to different activities such as carrying load, having repetitive movements on the hand, and working with vibratory tools, the prevalence of CTS among apprentice construction workers was 8.2% in the U.S. (11). The possible reason for the difference may be the level of development and strengths of occupational health and safety services.

Furthermore, in a study involving 200 construction apprentices in Hungary, no cases of CTS were found among apprentices (28). The possible reason for the difference may be that the average age of the Hungarian apprentices was 17 years and those in U.S.A was 27, and in our study, the average age was 30, which is 13 and 3 years, respectively, lesser than the population in our study. The very young age and lack of occupational exposure to wrist intensive activities among the Hungarian workers likely accounted for the absence of CTS.

On the contrary, 11.7% of construction workers doing manual work were diagnosed with CTS, and the prevalence rate is less than that found in France (19.7%) (29). This difference may be due to their larger sample size they used (1,275 participants) (mean age: 38.2 years). In addition, the prevalence rate in Egypt was 27.6%, and the possible justification for the difference may be due to their operational definition variation with this study. We used the Katz hand diagram positive findings in at least two of four clinical tests, the four flexion and compression test, carpal compression test, Phalen’s test, and Tinel’s test, and both occupational exposure and age are likely factors that account for differences among workers in these countries (30).

The findings of this study show that the age of the participants is significantly associated with carpal tunnel syndrome, as construction workers above 45 years of age were more likely to have carpal tunnel syndrome. This finding supported by the study carried out in Thailand (31). The possible explanation may be there is histological change in the transverse carpal ligament with age increase (become older) (32).

Participants who had a history of cigarette smoking were more likely to develop CTS than other participants who had no history of cigarette smoking. This finding was similar to a study conducted in Iran. In addition, Nathan et al. found a significant correlation between cigarette smoking and CTS (10, 27). The reason for this correlation could be that smoking can impair the vascular supply of the median nerve, thereby potentially increasing the susceptibility of the nerve to physical workloads, and prolonged tissue ischemia due to smoking may lead to median nerve degeneration and fibrosis (33).

Work experience of over 5 years among construction workers significantly increased the odds of carpal tunnel syndrome compared to those with less than 5 years of work experience. This finding is supported by a study conducted on 996 construction workers, where a longer duration of work has been found as a risk factor for CTS and corroborates with a study conducted in Egypt (14, 34, 35).

Furthermore, significant associations were observed between working with finger-pressing tools and carpal tunnel syndrome among construction workers (*p* < 0.023). The odds of working with finger-pressing tools were nearly five time more for the development of carpal tunnel syndrome than among those who were not working with finger-pressing tools among construction workers. This finding is supported by the review conducted in Canada (36), which suggests that the external load on the hand, especially on the fingers, significantly increases the pressure on the carpal tunnel (5). Overall, this finding is crucial for healthcare providers and policymakers in developing targeted interventions and preventive measures. It has the potential to make a significant contribution to understanding carpal tunnel syndrome (CTS) among construction industry workers in Gondar, Ethiopia and to inform strategies for its prevention, early detection, and management as an occupational health concern.

## Conclusion

The magnitude of carpal tunnel syndrome was 11.7% among construction workers in Gondar town. Older age, having more work experience, cigarette smoking, and working with finger-pressing tools were significantly associated with carpal tunnel syndrome among construction workers. Implementations of education programs that raise awareness about the risk of cigarette smoking along with encouragement from employers and supervisors to seek early medical intervention and treatment of carpal tunnel syndrome can help prevent the disease progression before they become a chronic problem. In Ethiopia, the reporting system for work-related degenerative conditions (CTS and LBP) is less robust and workers’ compensation programs are inadequate. Moreover, no compensations had been granted for the identified cases. However, the authors recommend that, in order to improve occupational health and safety standards, increased access to healthcare services and the strengthening of workers’ rights and compensation programs are essential for addressing work-related conditions such as CTS in low-income countries.

## Data availability statement

The original contributions presented in the study are included in the article/Supplementary material, further inquiries can be directed to the corresponding author.

## Ethics statement

The studies involving humans were approved by the Institutional Review Board of the University of Gondar College of Medicine and Health Science. The studies were conducted in accordance with the local legislation and institutional requirements. The participants provided their written informed consent to participate in this study.

## Author contributions

NB: Writing – original draft, Supervision, Project administration, Methodology, Investigation, Conceptualization. MG: Writing – review & editing, Project administration, Methodology, Investigation, Formal analysis, Data curation. SC: Writing – review & editing, Visualization, Validation, Software, Resources, Conceptualization. MM: Writing – original draft, Supervision, Methodology, Investigation, Formal analysis, Conceptualization. EY: Writing – original draft, Methodology, Formal analysis, Conceptualization.
