# Supplementary figures and images for: Burden of carpal tunnel syndrome and its associated factors among construction industry workers in Gondar town, Ethiopia
Source: Front Public Health. 2024 Jun 12;12:1365124. doi: 10.3389/fpubh.2024.1365124 (PMC11218597; doi:10.3389/fpubh.2024.1365124)

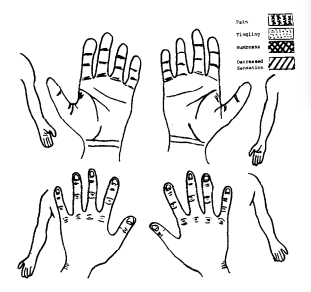


**Supplementary figure: 1 Kartz Hand diagram**

Supplement: Supplementary file 1 [file Data_Sheet_1.docx]
